# Hax-1 is required for Rac1-Cortactin interaction and ovarian carcinoma cell migration

**DOI:** 10.18632/genesandcancer.8

**Published:** 2014-03

**Authors:** Rohini Gomathinayagam, Jayaraman Muralidharan, Ji Hee Ha, Lakshmi Varadarajalu, Danny N. Dhanasekaran

**Affiliations:** ^1^ Stephenson Cancer Center and Department of Cell Biology, 975 NE 10^th^ Street, The University of Oklahoma Health Sciences Center, OK 73104

**Keywords:** Hax-1, Rac1, cortactin, cell migration, Metastasis, Ovarian Cancer

## Abstract

Hax-1 is a multifunctional protein, which is involved in diverse cellular signaling pathways including tumor cell survival and migration. We have shown previously that cell migration stimulated by the oncogenic G protein, G_13,_ requires Hax-1 for the formation of a functional complex involving Gα_13_, Rac1, and cortactin. However, the role of Hax-1 in cancer cell migration or its role in Rac1-cortactin complex formation, which is known to be required for such migration remains to be characterized. Results focused on resolving the role of Hax-1 in ovarian cancer pathophysiology indicate that Hax-1 is overexpressed in ovarian cancer cells and the silencing of Hax-1 inhibits lysophosphatidic acid (LPA)- or fetal bovine serum-stimulated migration of these cells. In addition, silencing of Hax-1 greatly reduces Rac1-cortactin interaction and their colocalization in SKOV3 cells. Mapping the structural domains of Hax-1 indicates that it interacts with cortactin via domains spanning amino acids 1 to 56 (Hax-D1) and amino acids 113 to 168 (Hax-D3). Much weaker interaction with cortactin was also observed with the region of Hax-1 spanning amino acids 169 – 224 (Hax-D4). Similar mapping of Hax-1 domains involved in Rac1 interaction indicates that it associates with Rac1 via two primary domains spanning amino acids 57 to 112 (Hax-D2) and 169 to 224 (Hax-D4). Furthermore, expression of either of these domains inhibits LPA-mediated migration of SKOV3 cells, possibly through their ability to exert competitive inhibition on endogenous Hax-1-Rac1 and/or Hax-1-cortactin interaction. More significantly, expression of Hax-D4 drastically reduces Rac1-cortactin colocalization in SKOV3 cells along with an attenuation of LPA-stimulated migration. Thus our results presented here describe for the first time that Hax-1 interaction is required for the association between Rac1 and cortactin and that these multiple interactions are required for the LPA-stimulated migration of SKOV3 ovarian cancer cells.

## INTRODUCTION

Cell migration is critical for both cellular homeostasis and development. It is regulated by a complex signaling network involving a coordinated interactions among large array of signaling and cytoskeletal structural proteins[1] [2, 3]. Aberrant cell migration has been shown to be associated with many different pathological conditions including cancer cell metastasis[4]. There has been intensive focus in the recent years to identify critical molecules that enable the invasive migration of cancer cells. In this context, it is of interest to note here that our previous studies have identified a pivotal role for Hax-1 in the migration of neoplastically transformed NIH3T3 cells[5]. Hax-1 was initially identified as an anti-apoptotic protein that interacts with Hematopoetic Cell-specific Lyn Substrate-1 (HCLS1/HS-1) [6]. The observations that HCLS1/HS-1 is a hematopoietic cell-specific homolog of cortactin and HCLS1/HS1 as well as cortactin is critically involved in cell migration [7-9] suggested a role for Hax-1 in cell migration. Subsequent studies from several laboratories including ours have clearly established a crucial role for Hax-1 in cancer cell migration [5, 10-12]. More recently it has been shown that Hax-1 is overexpressed in many cancers and the increased expression of Hax-1 can be correlated with aggressive cancer metastasis[13-20]. Collectively these studies point to a novel, but yet to be resolved role for Hax-1 in tumor cell migration and metastasis.

Our previous studies focused on identifying the role of Hax-1 in invasive cell migration regulated by the oncogenic heterotrimeric G protein, G_13_, have indicated the possible role of Hax-1 in facilitating the formation of a multi-protein complex consisting of Gα_13_, cortactin, and Rac1[5]. Several studies including ours have characterized the molecular basis for the interaction of Hax-1 with different proteins including Gα_13_ and cortactin. However, the structural and functional requirement for Hax-1 in Rac1-cortactin mediated signaling machinery involved in cell migration - especially with reference to cancer cell migration and metastasis - has not been fully understood. The most critical spatiotemporal cytoskeletal events regulated by Rac1 are membrane ruffling[21] and lamellipodia formation[22, 23]. Essentially, Rac1 stimulates the formation of lamellipodia at the leading edges of migrating cells by coordinating F-actin and Arp2/3 interaction[21-24]. A critical mechanism by which Rac1 mediates this process is through the translocation of cortactin to the cell periphery where cortactin promotes the nucleation of Arp2/3-F-Actin interaction as well as the stabilization of the assembled Arp2/3-F-actin network and lamellipodia[7, 9, 25-28]. Thus, the dynamic interaction between Rac1 and cortactin plays a pivotal role in cell migration [9, 29-33]. However, the possible role of any scaffold protein that can modulate the dynamic interaction between Rac1 and cortactin is thus far not clarified. Based on our previous findings that Hax-1 is in complex with Rac1 as well as cortactin, it can be hypothesized that Hax-1 facilitates the interaction between Rac1 and cortactin, thus functioning as a scaffold protein for their interaction. In testing this hypothesis, we report here that Hax-1 shows an increased expression in ovarian cancer cells and silencing of Hax-1 critically impairs the migration of the representative SKOV3 cells in response to LPA. We also show that Hax-1 is required for the interaction between Rac1 and cortactin. Focusing on identifying the domain(s) of Hax-1 required for its interactions with cortactin and Rac1, we demonstrate here that the interaction between Hax-1 and cortactin primarily involves two domains named Hax-D1 and Hax-D3 spanning amino acids 1 to 56 and amino acids 113-168 respectively. A weaker cortactin-interacting domain of Hax-1 (Hax-D4) defined by amino acids 169 to 224. Hax-1 interaction with Rac1 involves two distinct domains of Hax-1 that spans amino acids 57-112 (Hax-D2) and 169-224 (Hax-D4). Our results also indicate that the competitive inhibition of the interaction between endogenous Hax-1 and Rac1 or Hax-1 and cortactin by the ectopic expression of any of these domains led to a decrease in LPA-stimulated migration of ovarian cancer cell line SKOV3. More significantly, the expression of Hax-D4, which shows interaction with both Rac1 and cortactin, acutely inhibited LPA-stimulated colocalization of Rac1 and cortactin thereby attenuating LPA-stimulated migration of these cells. Thus, the results presented here define a critical scaffolding role for Hax-1 in LPA-stimulated Rac1-cortactin interaction and subsequent ovarian cancer cell migration.

## RESULTS

### Silencing of Hax-1 attenuates LPA-mediated migration of SKOV3 cells.

**Figure 1:**
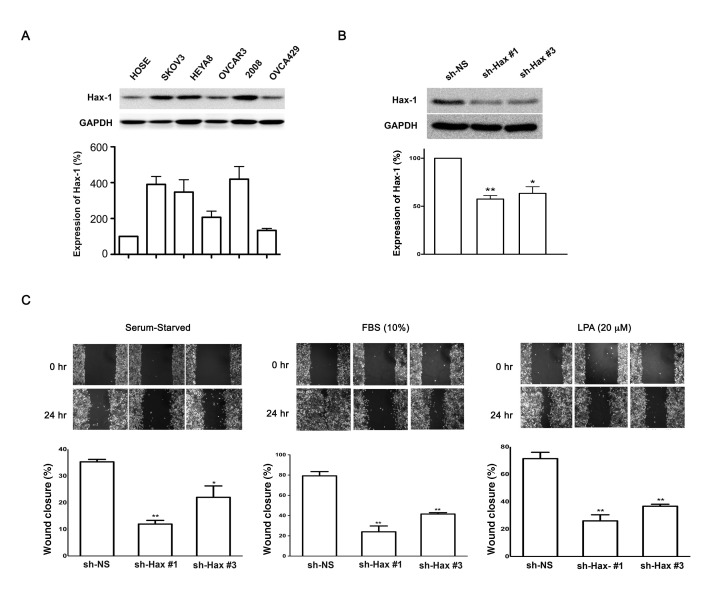
Silencing of Hax-1 attenuates LPA and FBS stimulated migration of SKOV3 cells. (A) Lysates (25 μg) from HOSE, SKOV3, HEYA8, OVCAR3, 2008, and OVCA429 ovarian cancer cells were collected, separated by 10% SDS-PAGE and subjected to immunoblot analysis with antibodies specific to Hax-1 or GAPDH (loading control). Expression levels of Hax-1 were quantified, normalized for the loading control (GAPDH), and the results were plotted as percent increase over the expressions levels seen in HOSE cells (mean ± SEM; n = 3). (B) Two transiently transfected shRNA constructs with Red fluorescent protein tag (RFP) for Hax 1(shHax #1, shHax #3) along with control cells that express non-specific shRNA with RFP (sh-NS) were assessed for Hax-1 expression using immunoblot analysis with antibodies to Hax-1. The blots were then stripped and reprobed with antibodies to GAPDH to monitor equal loading of protein. Expression of Hax-1 was quantified and presented as percent change over Hax-1 levels from control cells that express non-specific shRNA (mean ± SEM; n = 3). Statistical significance was assessed using One tailed t-test (* p <0.05 and ** p<0.001). (C) To determine the migratory potential, 1×10^6^ cells, plated in 35 mm plates were allowed to adhere overnight, serum deprived for 16 hours, and treated with 0.5 μM Mitomycin-C to arrest cell division. A linear scratch wound was made across the cell monolayer and cells were stimulated with 10% FBS or 20 μM LPA. Fields of view (10X) were selected at random, photographed and marked for re-identification. The identical fields were re-imaged following 24 hours of incubation and the images presented are representative of three independent experiments, each performed with triplicate fields of view. The percentage of migration in the serum starved (0.2% BSA), 10% FBS stimulated and 20 μM LPA treated groups were calculated in comparison with the control (mean ± SEM; n = 3). Statistical significance was assessed using One tailed t-test. * p <0.05; ** p<0.001.

**Figure 2:**
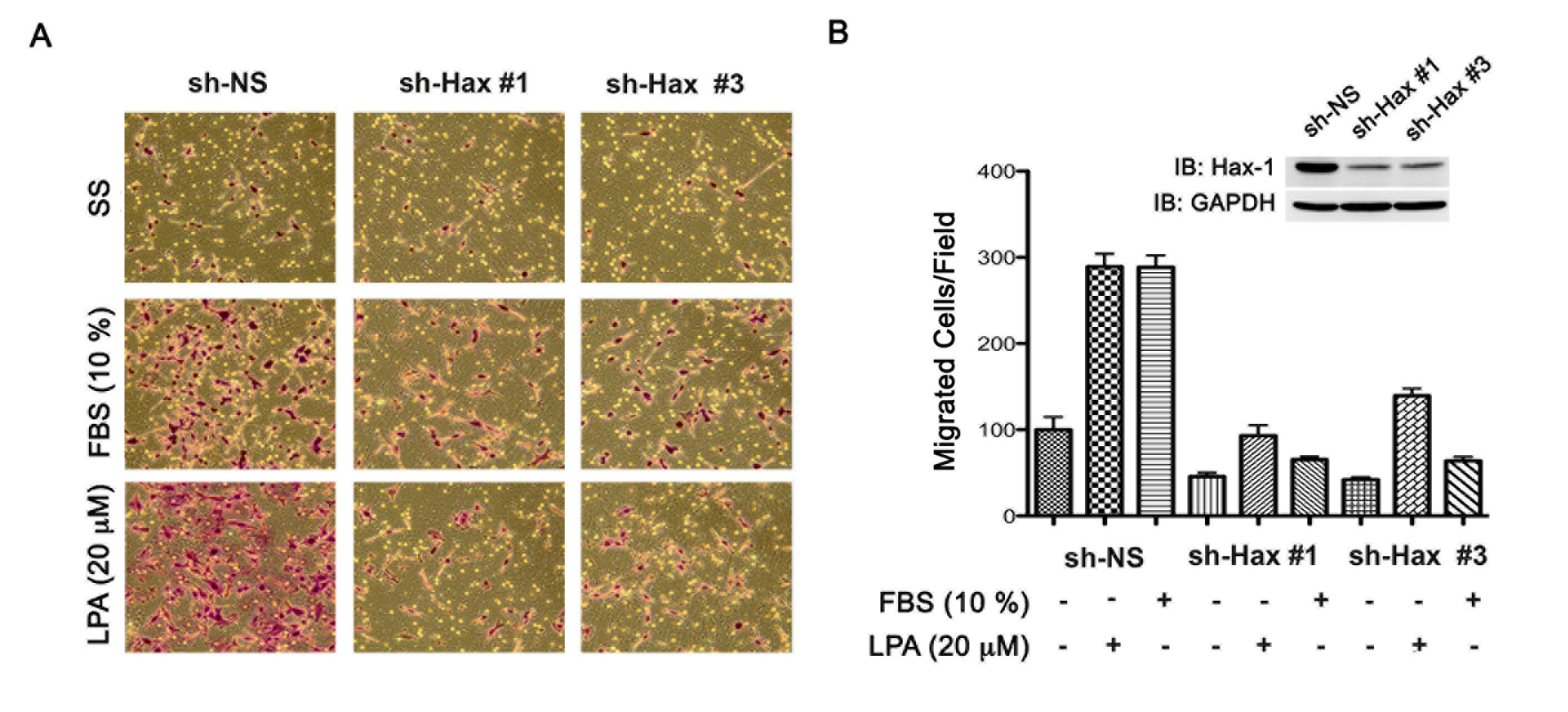
Silencing of Hax-1 attenuates LPA and FBS stimulated invasive migration of SKOV3 cells. (A) Transient silencing of Hax-1 was carried out by transfecting SKOV3 cells with vectors encoding shRNA specific for Hax-1 (sh-Hax 1 & sh-Hax #3) or scrambled shRNA control for 48 hours (sh-NS). These transfectants were unstimulated (SS), stimulated with LPA (20 μM), or FBS (10%) and their migratory responses were monitored as described under Materials and Methods. At 24 hours following stimulation, images were obtained from random fields of view at 10X magnification. The images shown are representative of three independent experiments, each performed with triplicate fields of view. (B) Cell migration profiles were quantified by enumerating the migrated cells in a minimum of three different fields. Results are presented as the number of migrated cells per field and the bars represent mean ± SEM from there independent experiments. Silencing of endogenous Hax-1 was monitored by immunoblot analysis using antibodies to Hax-1 (inset). The blot was stripped and reprobed with antibodies to GAPDH to monitor equal loading of protein.

Our previous studies have shown that Hax-1 is critically required for the invasive cell migration stimulated by the *gep* protooncogene, Gα_13_ [5]. Our studies have also demonstrated that LPA-stimulated Gα_13_ promotes the migration of cancer cell lines including those of ovarian cancer [34, 35]. Therefore, we first sought to investigate whether the expression of Hax-1 is increased in ovarian cancer cells in which Gα_13_-signaling plays a major role in invasive cell migration. Lysates from a panel of ovarian cancer cells including SKOV3, HeyA8, OVCAR3, 2008, OVCA429 cells and control human ovarian surface epithelial cells (HOSE) were subjected to immunoblot analysis using antibodies specific to Hax-1. Results from such an analysis clearly indicated that the expression of Hax-1 was increased in ovarian cancer cell lines compared to HOSE cells (Figure 1A). The elevated levels of expression of Hax-1 seen in ovarian cancer cells along with its previously established role on cell migration prompted us to investigate the role of Hax-1 in LPA or FBS stimulated migration of ovarian cancer cells. This was carried out using SKOV3 cells in which the expression of Hax-1 was transiently silenced. Two shRNA constructs, sh-Hax #1 and sh-Hax #3 that could efficiently silence Hax-1 were chosen for these analyses (Figure 1B). Equal number of SKOV3 cells (1×10^6^), expressing sh-Hax #1, sh-Hax #3, or scrambled, non-specific shRNA-control RFP vector, were subjected to a standard “wound-healing” assay in the presence of 20 μM LPA, or 10% FBS along with appropriate controls. The results indicated that the silencing of Hax-1 drastically inhibited LPA- or serum-stimulated migration of SKOV3 cells compared to the control cells (sh-NS) expressing scrambled shRNA (Figure 1C). To test the role of Hax-1 in LPA- or serum-stimulated invasive migration of these cells, we monitored the migration of Hax-1-silenced SKOV3 cells using a Collagen I-coated TransWell invasion assay. Similar to the results obtained from the wound-healing assay, LPA- as well as FBS-stimulated invasive migration of ovarian cancer cells was significantly attenuated by the silencing of Hax-1 (Figure 2 A, B). Together, these data establish a dominant role for Hax-1 in LPA stimulated invasive migration of ovarian cancer cells.

### Silencing of Hax-1 disrupts Rac1- cortactin interaction and localization

**Figure 3:**
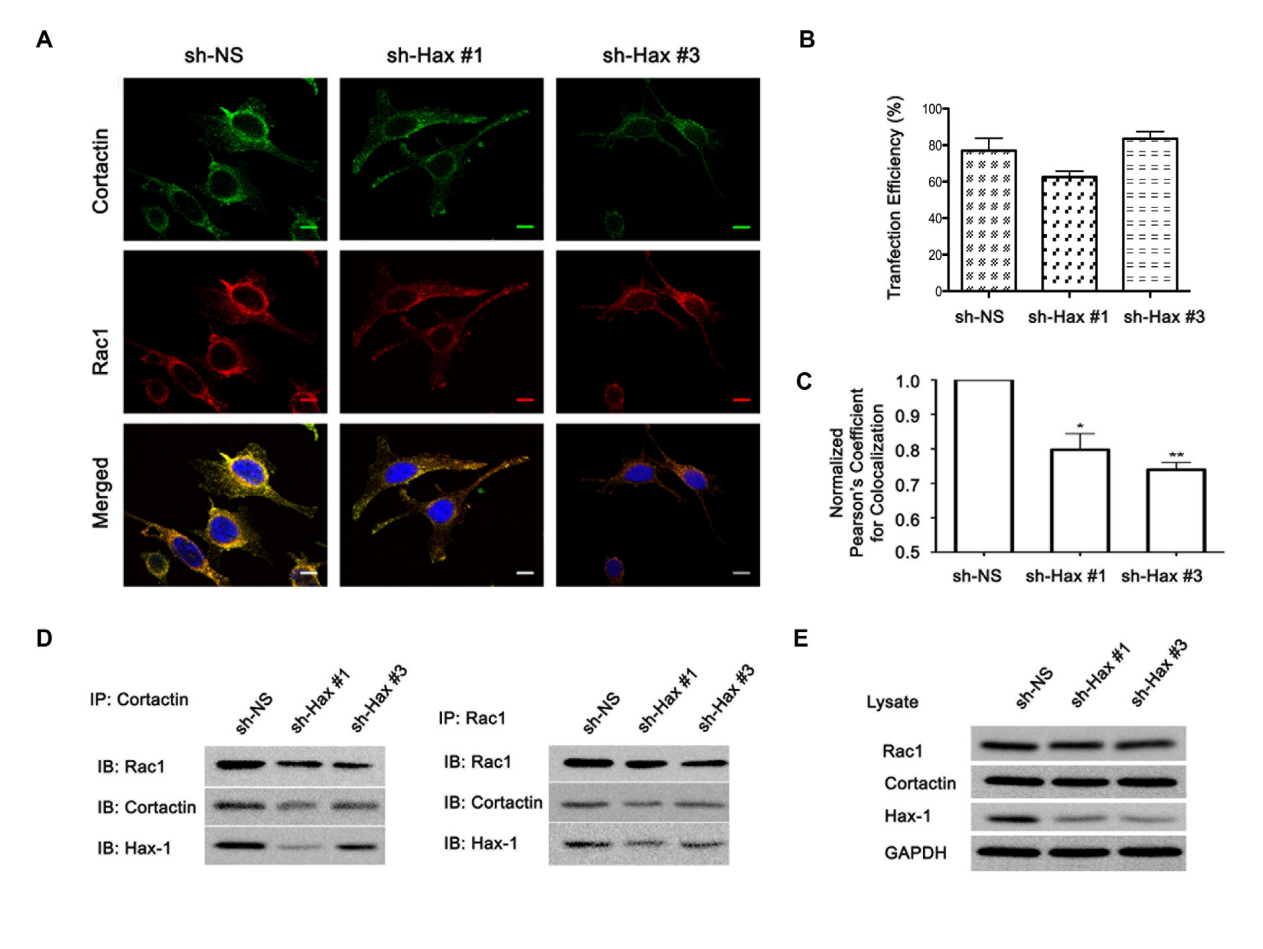
Silencing of Hax-1 attenuates Rac1-cortactin complex formation. (A) SKOV3 cells in which the expression of Hax-1 was silenced by shRNA to Hax-1 (sh-Hax #1 and sh-Hax #3) and the control cells expressing non-specific shRNA (shNS) were sequentially immunostained with primary mouse monoclonal Rac1 (1:200) antibody for 1h, washed, incubated with secondary Alexa 647-conjugated goat anti-mouse IgG (1:200, Red) for 1h washed and then stained with Alexa 488-conjugated anti-mouse Cortactin (Millipore, MA) antibody for 1h (1:200, Green). Fluorescent micrographs were collected with a Leica SP2 MP Confocal microscope using 63x Plan APO 1.4 NA oil immersion objective. Colocalization of Rac1 and cortactin (Yellow) were analyzed, on 3 images for each condition per experiment, using NIH ImageJ software. The scale bar 20 μm is common for all channel images. In order to determine the transfection efficiency/ silencing efficiency for Hax-1, the RFP-tagged shRNA transfected cells were fixed with 3% paraformaldehyde and the nuclei were DAPI stained. RFP and DAPI fluorescent micrographs were collected with a Nikon TE2000-E inverted microscope using 20x Plan Fluor NA 0.45 dry objective. (B) Using NIH ImageJ software, numbers of transfected and untransfected cells were counted. The transfection efficiency was calculated as the ratio of transfected to untransfected cells and the graph represents the percentage of transfected cells. (C) For the quantitative determination of the Rac1-cortactin colocalization, Pearson's correlation coefficient (PCC) calculated using JACoP (Just Another Colocalization Plugin) for each transfection condition was normalized to the vector control transfection. Average of the normalized PCC is represented as a graph and the statistical significance was assessed using one-tailed t-test (*p <0.1 and ** p<0.05). (D) SKOV3 cells (1×10^6^) were transiently transfected with vectors encoding control shRNA (sh-NS) or shRNA specific to Hax-1 (sh-Hax #1 and Hax #3). At 48 hours following transfection, 1mg of the cellular proteins was immunoprecipitated with cortactin (*Left Panel*) or Rac1 (*Right Panel*) antibodies. Immunoprecipitates were resolved by SDS-PAGE and immunoblot analysis was carried out to detect the coimmunoprecipitation of Rac1 or cortactin respectively along with Hax-1. (E) 25 μg of the cell lysates served as input controls and were assessed for the expression levels of Rac1, cortactin, and Hax-1. Reprobing the blots with GAPDH antibody served to monitor equal loading of protein. The experiments were repeated at least three times and the presented results are from a typical experiment.

Our previous studies have indicated that Hax-1 is part of a signaling complex consisting of Gα_13_, cortactin, and Rac1[5]. It is well established that Rac1 plays a critical role in cell migration through its interaction with cortactin [8, 9, 36]. Upon stimulation by serum or specific ligands, it has been observed that Rac1 interacts with cortactin and the resultant Rac1-cortactin complex translocates to the leading edges of migrating cells to stabilize Arp2/3-actin nucleation complex involved in lamellipodia formation [9, 26, 28]. Based on our previous finding that, Hax-1 exists in a complex with Rac1 as well as cortactin, both of which coimmunoprecipitate with Hax-1, it can be reasoned that Hax-1 is involved in promoting Rac1-cortactin interaction. To verify such a role for Hax-1, we examined the colocalization of Rac1 and cortactin in Hax-1-silenced SKOV3 cells using immunofluorescence analysis. As shown in Figure 3A, a robust interaction between Rac1 and cortactin could be seen in control SKOV3 cells (sh-NS). Whereas, cells in which Hax-1 was silenced exhibited a significantly reduced colocalization of Rac and cortactin (Figure 3A). Quantification of colocalization substantiated this further (Figure 3C). We also carried out coimmunoprecipitation analysis using the lysates from these cells, to test whether the silencing of Hax-1 leads to a reduction in the physical association between Rac1 and cortactin. As shown in Figure 3D, the levels of Rac1 coimmunoprecipitated with cortactin were greatly reduced in lysates from Hax-1-silenced SKOV3 cells compared to the control cells. Similarly, the levels of cortactin coimmunoprecipitated with Rac1 were reduced in Hax-1 silenced cells. The observation that the expression levels of Rac1 and cortactin remained unchanged with the silencing of Hax-1 indicated that the reduced levels of coimmunoprecipitated Rac1 and cortactin were not due to changes in the expression levels of either of these proteins. Together, these results (Figure 3A-C) demonstrate a novel role for Hax-1 in facilitating the interaction between Rac1 and cortactin.

### Hax-1 interacts with cortactin and Rac1 via distinct regions

**Figure 4:**
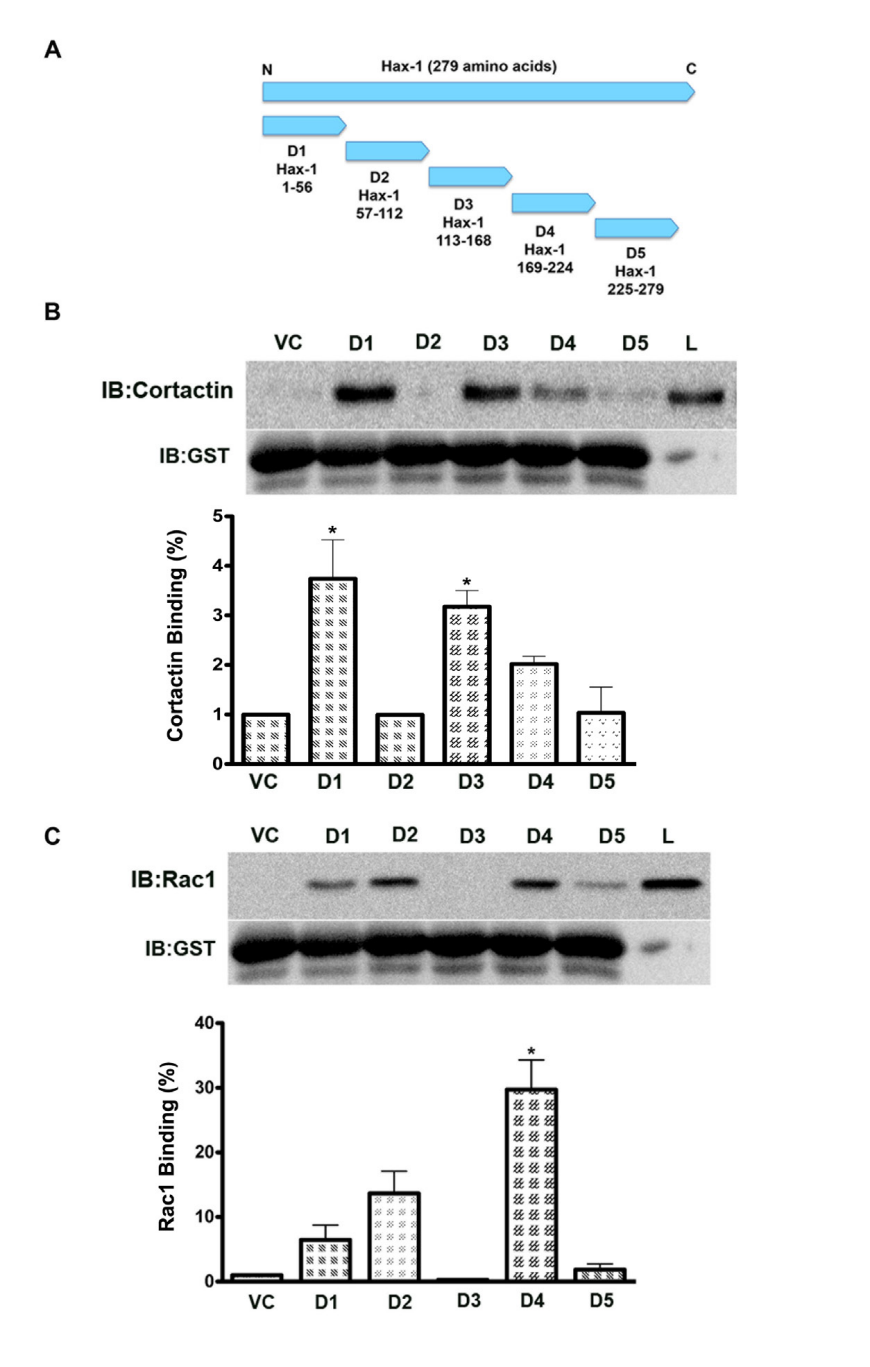
Hax-1 interacts with cortactin and Rac1 via distinct regions. (A) Hax-D1, Hax-D2, Hax-D3, Hax-D4, and Hax-D5 domain constructs, labeled D1, D2, D3, D4, and D5, comprising of 56 consecutive amino acids, were cloned by PCR methods in pGEX 5x-1 vector. (B, C) The GST-D1-D5 Sepharose beads were prepared using protocols as discussed under Materials and Methods section from the GST-Hax-D1-D5 domain constructs. 2 mg of SKOV3 lysate protein obtained using previously published procedures were incubated with the GST- D1, GST-D2, GST-D3, GST-D4, and GST-D5 Sepharose beads for 4 hours at 4°C. The beads were washed five times with chilled GST-lysis buffer, and eluted in SDS sample-loading buffer. The co-precipitated Rac1 or cortactin along with the putative interacting domain, with Vector control (VC) and lysate input control (L) were identified by immunoblot analysis. Results presented are from a typical experiment and each experiment was carried out at least thrice. Binding percentage of cortactin (Figure 4B) and Rac1 (Figure 4C) were assessed and the error bars are presented as mean ± SEM. The statistical significance was assessed using one-tailed t-test. * p<0.05.

The observation that the silencing of Hax-1 led to a reduction in the levels of colocalized Rac1 and cortactin underscored the need to define the interaction of Hax-1 with these proteins further. Our previous studies have shown that the immunoprecipitation of an epitope-tagged full-length Hax-1 brings down both cortactin and Rac1[5]. Therefore, we carried out studies to identify the domains of Hax-1 involved in the interaction with cortactin and Rac1 using a GST-fusion protein pull-down assay strategy. A sequential series of cDNA inserts encoding fifty-six amino acids of Hax-1 (Figure 4A) were ligated to a cDNA insert encoding GST protein by PCR methods and were utilized for the expression of GST-fused domains of Hax-1 in E. coli. GST-Hax-1-Sepharose beads generated from these constructs (Figure 4A), namely Hax-D1 (amino acids 1-56), Hax-D2 (amino acids 57-112), Hax-D3 (amino acids 113-168), Hax-D4 (amino acids 169-224), and Hax-D5 (amino acids 225-279), were used to pull down cortactin or Rac1 from the lysates prepared from SKOV3 cells. As shown in Figure 4B, two major cortactin-interaction domains of Hax-1 could be mapped to amino acids 1-56 (Hax-D1) and 113-168 (Hax-D3). In addition, a much weaker interaction site was mapped to a site spanning amino acids 169-279 (Hax-D4). Analyses of GST-Hax-1-Rac1 interactions indicated that the major interaction sites for Rac1 were amino acids 57-112 (Hax-D2) and 169-224 (Hax-D4). Relatively weaker interaction sites for Rac1 could be mapped to other regions of Hax-1 spanning amino acids 1-56 (Hax-D1) and 225-279 (Hax-D5) (Figure 4C). Thus, the results presented here identifies for the first time that Hax-1 interacts with cortactin and Rac1 via specific as well as overlapping domains.

### Expression of Cortactin- and Rac1-interacting domains inhibits the migration of SKOV3 Cells

**Figure 5:**
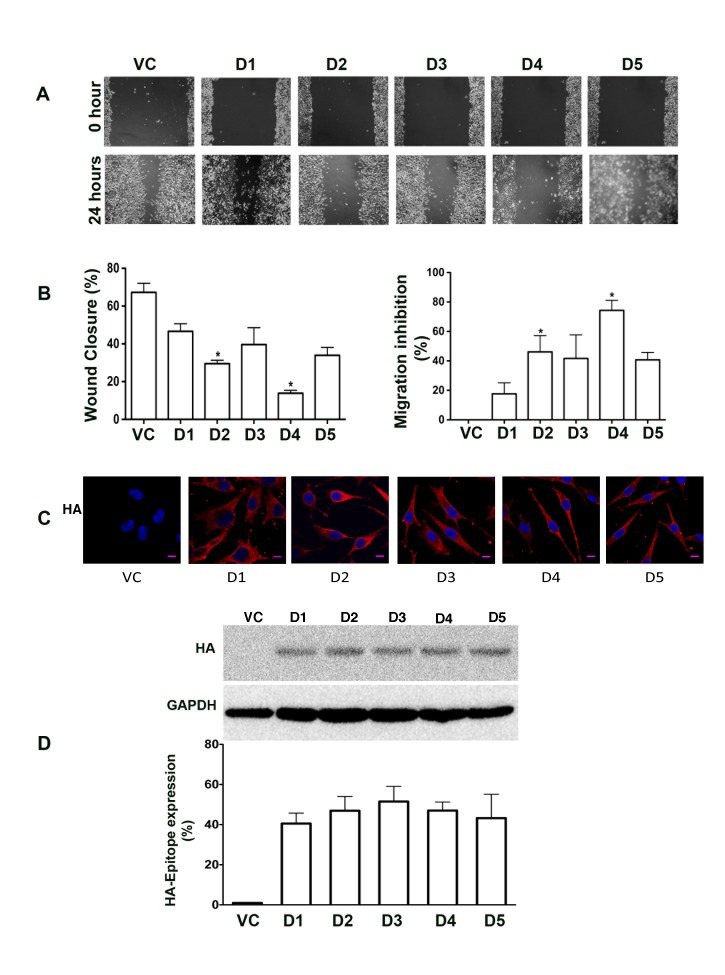
Expression of Rac1-interacting domains of Hax-1 inhibits the migration of SKOV3. (A) SKOV3 control cells transfected with the empty pcDNA3.1+ vector (VC) and SKOV3 cells transiently transfected with HA-epitope tagged domains of Hax-1, Hax-D1, Hax-D2, Hax-D3, Hax-D4, and Hax-D5 (denoted as D1, D2, D3, D4, and D5 respectively), were utilized to analyze the role of Hax-1 domains on cell migration. At 24 hours after transfection, 0.5 μM Mitomycin-C was added to the transfectants (5×10^5^/dish) to prevent cell division. Linear scratch wounds were made with 200 μl pipette tips across the monolayer of cells in the respective dishes to initiate the wound-healing assay. At 0 hour, fields of view (10X) were selected at random, photographed and marked for re-identification. The identical fields were re-imaged after 24 hours of incubation. The images presented are representative of three independent experiments, each performed with triplicate fields of view. (B) Percentage of wound closure and the migration inhibition percentage were calculated based on the migration of the transfectants expressing vector control and the respective domains. The statistical significance was assessed using students-t test. * p<0.05. Error bars are presented as mean ± SEM for triplicate experiments. (C) Expression of the respective Hax-1 domains was monitored by immunostaining with HA-epitope antibody. Three different fields were viewed and three independent experiments were performed. Fluorescent micrographs were collected with a Leica SP2 MP Confocal microscope using 63x Plan APO 1.4 NA oil immersion objective. The results presented here are from a typical experiment and the scale bar is 10 μm for all the images. (D) Expression of HA-tagged Hax-D1-D5 domains (labeled as D1, D2, D3, D4, D5) were monitored along with vector control (VC) by immunoblot analysis using lysates (50 μg) derived from the respective transfectants. Lysates were resolved in 15% SDS-PAGE gels and immunoblotted with an antibody against HA-epitope. The blot was stripped and reprobed with GAPDH antibody to monitor equal loading of protein. The HA-epitope reactive bands were quantified and plotted as percent expression over GAPDH levels (mean ± SEM; n = 3).

Results from the *in vitro* binding assays described above indicate that Hax-1 interacts with both Rac1 and cortactin through non-overlapping as well as overlapping domains. Therefore, it can be reasoned that Hax-1 brings together Rac1 and cortactin through this interaction during cell migration. In such an event, the expression of cortactin- or Rac1-interacting Hax-1-domain would inhibit cell migration by competing with the endogenous Hax-1 in its interaction with cortactin and/or Rac1. To investigate, we analyzed the migratory potential of SKOV3 cells expressing the different Hax-1 domains. As shown in Figure 5, expression of these domains blunted the migratory potential of SKOV3 cells by varying levels (Figure 5A, 5B). However, more pronounced inhibitory effect on cell migration was observed with the expression of the Hax-D4. The inhibitory effects of Hax-D4 on migration were quantified to be > 75% (Figure 5A, 5B). We confirmed the equal expression of the HA-tagged Hax-1 domains by immunostaining (Figure 5C) and immunoblotting for the HA-epitope along with appropriate quantification (Figure 5D).

### Hax-D4 Domain Potently Inhibits Rac1-cortactin Interaction.

**Figure 6:**
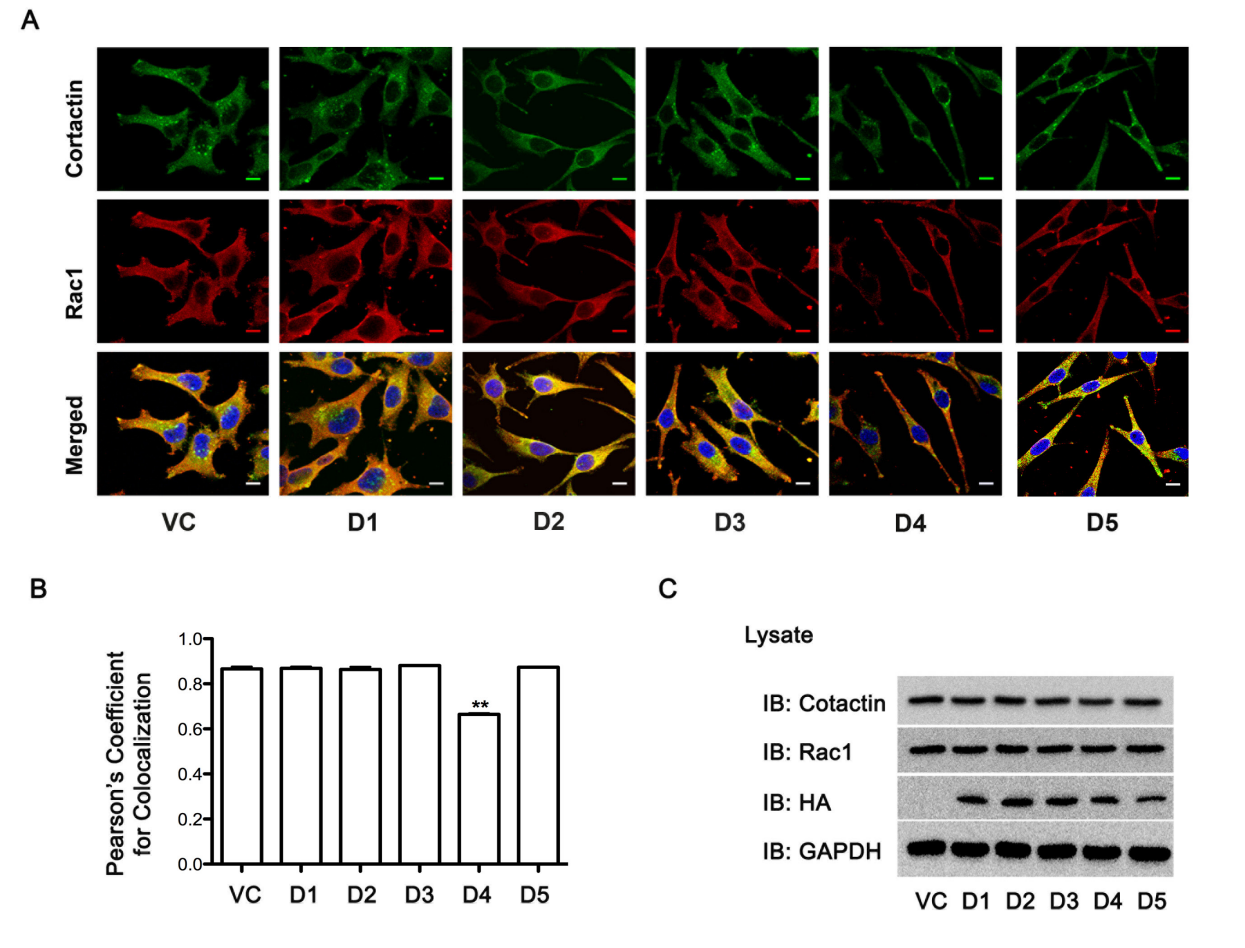
Expression of Rac1-interacting D4 domain of Hax-1 attenuates Rac1-cortactin complex formation. (A) pcDNA3-SKOV3 vector control cells (VC) and pcDNA3 Hax D1-D5 domain transfected cells (D1, D2, D3, D4, D5) were immunostained with primary mouse monoclonal Rac1 (1:200) antibody for 1h, washed, incubated with Alexa 568-conjugated goat anti-mouse IgG for 1h (Red), washed and then stained with Alexa 488-conjugated anti-mouse Cortactin (Millipore, MA) antibody for 1h (Green). Fluorescent micrographs were collected with a Leica SP2 MP Confocal microscope using 63x Plan APO 1.4 NA oil immersion objective. Colocalization of Rac and Cortactin (Yellow) were analyzed, on 3 images for each condition per experiment, using NIH ImageJ software. The scale bar 10 μm is common for all images. (B) For the quantification of Rac1-cortactin colocalization, Pearson's correlation coefficient (PCC) calculated using JACoP (Just Another Colocalization Plugin) for each transfection condition was normalized to the vector control transfection and the average of the normalized PCC is represented as a graph (Figure 6-side panel, lower figure). The statistical significance assessed using one-tailed t-test. ** p<0.05. (C) Lysates (25 μg) from cells transfected with vector-control or vectors encoding Hax-D1-D5 domains were subjected to immunoblot analysis to monitor the expression of HA-tagged Hax-1 domains (D1, D2, D3, D4, and D5), endogenous Rac1, cortactin using the respective antibodies. Equal loading of the lysates was monitored by reprobing the blots with GAPDH antibody. The experiments were repeated at least three times and the presented results are from a typical experiment.

It is well established that the interaction of Rac1 with cortactin is an early event in lamellipodia formation [9, 37]. Therefore, we investigated whether the ectopic expression of the distinct domains of Hax-1 would inhibit such interaction. SKOV3 cells transfected with vectors encoding different domains of Hax-1 were subjected to immunofluoresence analysis for the presence of Rac1 and cortactin. The extent of colocalization of Rac1 and cortactin did not show a significant change in cells expressing Hax-D1, Hax-D2, Hax-D3, or Hax-D5-domains of Hax-1 compared to the vector control cells (VC). However, cells expressing Hax-D4 showed a significant reduction (> 30%) in the colocalization of cortactin and Rac1 (Figure 6A & B).

### Hax-D4 domain inhibits LPA-stimulated Rac1-cortactin interaction and cell migration.

**Figure 7:**
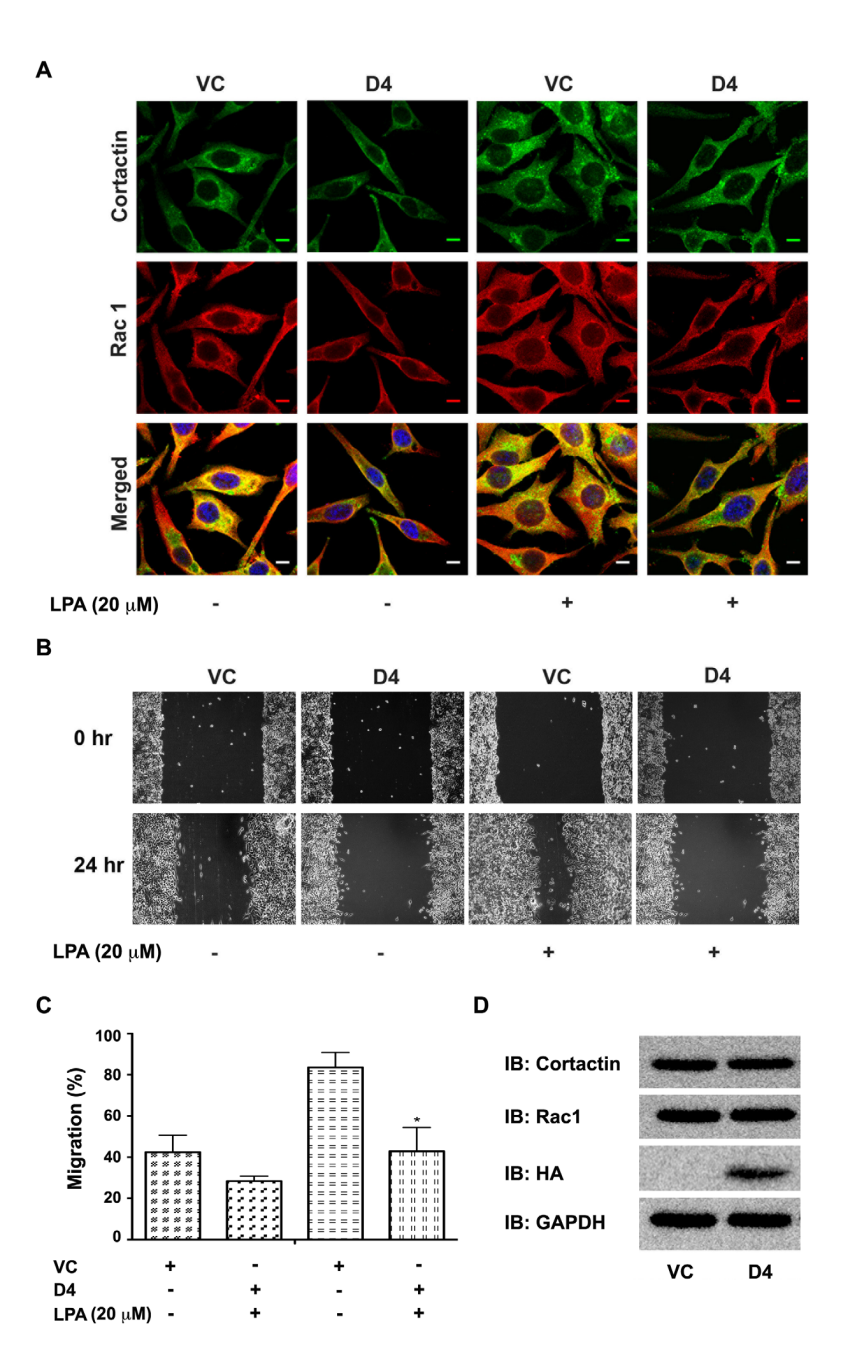
Expression of Hax-D4 attenuates LPA stimulated Rac1-cortactin complex formation in SKOV3 Cells. (A) pcDNA3-SKOV3 vector control cells (VC) and pcDNA3-Hax D4 domain transfected cells (D4) were serum starved for 16 hours and stimulated with 20 μM LPA for 24 hours. At the end of 24 hours, the cells were immunostained for Rac 1 and cortactin following previously discussed procedures. Fluorescent micrographs were collected with a Leica SP2 MP Confocal microscope using 63x Plan APO 1.4 NA oil immersion objective. Colocalization of Rac and Cortactin (Yellow) were analyzed, on 3 images for each condition per experiment, using NIH ImageJ software. The scale bar 10 μm is common for all images. (B) SKOV3 cells were transiently transfected with pcDNA3-SKOV3 vector control (VC) and pcDNA3-Hax D4 domain. 24 hours after transfection, transfectants were serum starved for 16 hours and linear scratch wounds were made across the monolayers to assay the wound healing efficiency of the cells. Random fields of view (10X) were photographed, marked for re-identification and stimulated with 20 μM LPA and identical fields were re-imaged following 24 hours. The experiments were performed thrice independently and presented images are from a typical experiment. (C) The percentage of wound closure was calculated based on the migration of the control and the D4 domain transfected cells (mean ± SEM; n = 3). The statistical significance was assessed using one-tailed t-test. * p<0.05. (D) 50 μg of lysate protein from D4 domain transfected cells was resolved using 15% SDS-PAGE and immunoblot analysis was carried out with antibodies against Rac1, cortactin and HA-epitope. The blot was stripped and reprobed with an antibody to GAPDH for ensuring equal protein loading.

Previous studies have shown that lysophosphatidic acid (LPA) plays a major role in ovarian cancer pathobiology by promoting the invasive migration of ovarian cancer cells [34, 38-43]. Therefore, it can be predicted that LPA-stimulated migration involves Hax-1 mediated complex formation involving Rac1 and cortactin. To test, we monitored whether LPA stimulates the interaction between Rac1 and cortactin and if so, whether the expression of Rac1/Cortactin-interacting Hax-D4 would abrogate such LPA-mediated Rac1-cortactin association. SKOV3 cells transfected with control vector (VC) or vector encoding Hax-D4 were serum-starved for 16 hours, following which they were stimulated with 20 μM LPA for 24 hours. The transfectants were subjected to immunofluorescence imaging by probing them with antibodies specific to Rac1 or cortactin along with specific fluorophore-tagged second antibodies. Results from such analyses indicated that the cells stimulated with LPA showed an increase in Rac1-cortactin complexes, indicated by their broad colocalization (Figure 7A). More interestingly, the expression of the Hax-D4 drastically inhibited such complex formation stimulated by LPA (Figure 7A).

Based on the observation that Hax-D4 inhibits LPA-stimulated Rac1-cortactin colocalization, it can be predicted that the Hax-4 would inhibit LPA-stimulated migration. To test, SKOV3 cells (1×10^6^) expressing the control vector or Hax-D4 were subjected to a cell migration assay in the presence of 20 μM LPA along with appropriate controls. Results from such analysis indicated that while LPA stimulated the migration of vector control SKOV3 cells, the expression of Hax-D4- drastically inhibited the migration of SKOV3 cells in response to LPA (Figure 7B). To make sure that the observed inhibitory effect on Rac1-cortactin colocalization and cell migration is not due to an altered expression of Rac1 or cortactin, their expression profiles were monitored along with that of the ectopically expressed Hax-D4 (Figure 7C). Together with the findings that Hax-D4 can interact with both Rac1 and cortactin (albeit weakly), these results indicate that Hax-D4 disrupts LPA-stimulated Rac1-cortactin colocalization and cell migration by competitively inhibiting the interaction of Hax-1 with Rac1 and cortactin.

## DISCUSSION

Hax-1 was initially identified as HS-1 or HCLS1–interacting protein in a yeast two hybrid screen. Subsequent studies have identified its interaction with many different proteins involved in different cellular responses such as cell survival, anti-apoptosis, and cell migration. Recently, it has also been observed that the homozygous mutations in Hax-1 gene or loss of Hax-1 are associated with severe congenital neutropenia and neurodevelopmental disorders. In addition, overexpression of Hax-1 has been observed in tissues from esophageal, lung and colon cancers [13, 14, 17, 20]. More significantly, in colorectal cancer the increased expression of Hax-1 correlates with the lymph node metastasis and poor prognosis of the patients[13]. While all these studies point to a determinant role for Hax-1 in critical signaling pathways involved in cell growth, differentiation, apoptosis, tumorigenesis and tumor cell migration, the functional role played by Hax-1 in these diverse cellular responses are far from clear.

The findings that Hax-1 interacts with HS1 and cortactin[6] that are involved in cell migration[6, 7, 27, 44, 45] provided the initial evidence that Hax-1 could be involved in cell migration. In this context, our previous observation that Hax-1 is required for the migration of NIH3T3 cells stimulated by the activated, oncogenic mutant of Gα_13_ is highly significant. These studies suggested that Gα_13_, upon activation by its cognate receptor(s), associates with Hax-1 in a complex containing Hax-1, Rac1 and cortactin. Based on these findings, we proposed that Hax-1 could provide a physical conduit for transmitting the signals from the oncogenic G protein to cellular cytoskeletal machinery through its interactions with cortactin. While the structural basis for the association of Hax-1 with Gα_13_ and cortactin is well characterized, such an understanding has been lacking for the interaction between Hax-1 and Rac1. It has also remained unclear whether the interaction of Hax-1 with Rac1 would have any bearing on the Rac1-cortactin interaction involved in cytoskeletal changes associated with cell migration. Results from our studies presented here attempts to clarify these points in addition to providing novel insights into the role of Hax-1 in cell migration. We demonstrate here that the expression of Hax-1 is highly elevated in a panel of ovarian cancer cells and the silencing of Hax-1 inhibited basal as well as serum- or LPA-stimulated migration by more than 50%. The inhibition was more acutely observed when invasive migration of SKOV3 cells in which Hax-1 was silenced was monitored using collagen I coated TransWell invasion assay. Although a role for Hax-1 in metastasis has been speculated based on its expression profile in metastatic cells [13, 14, 17, 20], the results presented here provide the first evidence that Hax-1 is critically required for the invasive migration of an ovarian carcinoma cell line in response to both LPA and serum growth factors. In light of the findings that LPA plays a dominant role in ovarian cancer cell migration, the observation that LPA-stimulated migration requires Hax-1 is quite significant.

It should be noted here that the studies carried out in several laboratories, including ours, have established Hax-1 as a non-enzymatic protein that interacts with multiple protein partners in different cellular or physiological contexts. Thus, it appears that Hax-1 behaves more like a scaffold protein involved in the nucleation of different multi-protein complexes in a cellular or physiological context specific manner. Our interrogation of Hax-1 based on this reasoning clearly substantiates such a view. Results from coimmunoprecipitation analyses clearly indicate that Hax-1 is required for the physical association between Rac1 and cortactin. This is further supported by the *in vivo* colocalization analysis in which the silencing of Hax-1 drastically reduces the colocalization of Rac1 and Hax-1. Next, our analysis of Hax-1 domains involved in its interactions with Rac1 and cortactin clearly indicates that the primary domains of Hax-1 involved in its interactions with Rac1 and cortactin are different. Our studies on mapping the cortactin-interacting sites of Hax-1 further refines this, by indicating that the cortactin-interacting sites of Hax-1 are interspersed in the N-terminus. Two primary cortactin-interacting sites span amino acids 1-56 (Hax-D1) and 113-168 (Hax-D3). In addition, Hax-D4 domain defined by amino acids 169-224 shows a weaker interaction with cortactin. The weaker interaction of Hax-D4 with cortactin could be due to the possibility that the cortactin-interacting site of Hax-1 extends beyond Hax-D3 and into the contiguous sequences of Hax-D4. In the case of Rac1 interaction, the primary sites through which Hax-1 interacts with Rac1 involve amino acids 57-112 (Hax-D2) and 169-224 (Hax-D4). Although the folding of these domains in relation to the tertiary structure of Hax-1 is not known at present, the non-overlapping sites (Rac1-specific Hax-D2 and cortactin-specific Hax-D3) involved in cortactin and Rac1 interaction suggest the interesting possibility that Hax-1 can interact with both the molecules using different domains so that they are brought into close proximity with each other for functional interaction. Further analyses using shorter deletion constructs or mutational scanning should provide more details on Rac1- and cortactin-specific domains of Hax-1.

**Figure 8:**
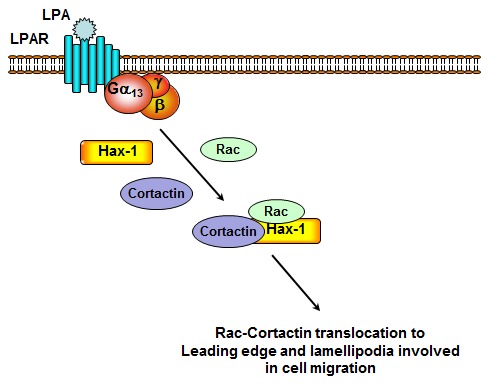
Schematic model for the scaffolding function of Hax-1. LPA, upon binding to one or more LPA receptors, activates Gα_13_ to stimulate the invasive migration of carcinoma cell lines [34, 35]; Gα_13_ in turn stimulates Hax-1[5] and Hax-1, thus stimulated by the upstream LPA-LPAR signaling promotes the interaction of Rac1 and cortactin through specific domains (*see text*). Hax-1 mediated interaction between Rac1 and cortactin is required for LPA-stimulated migration of the ovarian carcinoma cell line, SKOV3. The role of Hax-1 in subsequent molecular events during cell migration remains to be clarified.

It can be anticipated that the ectopic expression of Hax-1-domains that interact with either cortactin or Rac1, would inhibit cell migration by competitively inhibiting the interaction of endogenous Hax-1 with cortactin and/or Rac1. This view is substantiated by our studies in which the expression of cortactin- as well as Rac-interacting Hax-1-domains inhibited the migration of SKOV3 cells at varying extents. However, more potent inhibition of cell migration was observed with the expression of Hax-D4. When these domains were analyzed for their effect on Rac1-cortactin colocalization, only Hax-D4 was observed to acutely inhibit the colocalization. The inhibitory effect seen only with Hax-D4 could be due to the ability of Hax-D4 to interact with both Rac1 and cortactin. Although Hax-D4 shows a weaker interaction with cortactin compared to Rac1, it is likely that Hax-D4, through its bivalent interaction, competitively and effectively sequesters both Rac1 and cortactin, thus preventing them from interacting with each other. The fact that the observed inhibitory effects of Hax-D4 are quite similar to the effects seen with the silencing of Hax-1 (Fig. 1C), strongly supports the conclusion that the primary function of Hax-1 in cell migration is to facilitate the interaction of cortactin and Rac1 as a scaffolding protein. Taken together with our previous observations that LPA-stimulated migration of ovarian cancer cells require Gα_13_-regulated Rac-activation [34, 46] and activated Gα_13_ promotes the formation of a Hax-1-Rac1-cortactin signaling complex [5], the present observation that Hax-1 is required for Rac1-Cortactin interaction establish a critical role for Hax-1 in scaffolding the interaction between Rac1 and cortactin (Fig. 8). At present, it is not clear whether Hax-1 is required only for the nucleation of Rac1-cortactin interaction or if it is also required for the subsequent steps in cell migration including Rac1-mediated translocation of cortactin to lamellipodia and Rac1-cortactin-mediated stabilization of actin polymerization in lamellipodia. However, these speculated roles for Hax-1 need not be mutually exclusive. Further studies should define the processive events involving Hax-1, Rac1, cortactin and other binding partners during cell migration and metastasis. In summary, our results conclusively establish for the first time that Hax-1 is critically required for the Rac1-cortactin interaction and subsequent invasive migration of ovarian cancer cells.

## METHODS

### Cell culture and transfections

HOSE, SKOV3, HEYA8, OVCAR3, 2008, and OVCA429 ovarian cancer cells were cultured and maintained in Dulbecco's modified Eagle's Medium (DMEM) (Cellgro, Manassas, VA), containing 10% Fetal Bovine Serum (Gemini Bio-Products, West Sacramento, CA), 50 units/mL Penicillin, and 50 μg/mL Streptomycin at 37°C in a 5% CO_2_ incubator as described previously[34, 46, 47]. LPA was obtained from Avanti Polar Lipids (Alabaster, AL) and was dissolved to 20 mM stock solutions in sterile water and stored at −20°C. All transfections were carried out using Amaxa Biosystems Nucleofector II according to the instructions of the manufacturer.

### pRFP-c-RS shRNA constructs against HAX-1

pRFP-C-RS scrambled control shRNA construct (TR30015) and eight pRFP-C-RS shRNA constructs (TF312515) against HAX-1 with Chloramphenicol and Puromycin resistance markers were purchased from OriGene Technologies, Inc., Rockville, MD. The Hax-1 silencing efficiency of all the constructs were tested in SKOV3 cells and two shRNA constructs with efficient and target specific silencing (denoted by sh-Hax #1 and sh-Hax #3) were utilized for the experimental studies.

### pGEX 5X-1 GST D1-D5 constructs

GST fusion constructs encoding a sequential set of 56 amino acid domains from the N-terminus termed D1, D2, D3, D4, and D5, were constructed using a pGEX 5x-1 (Invitrogen) vector. Hax domains D1 to D5 were PCR amplified from pcDNA3.1 (+) full length Hemagglutinin-tagged-Hax-1 (HA-Hax-1) vector with primers containing appropriate restriction sites for cloning. While D1 was PCR amplified to contain EcoRI and Sal I restriction sites, D2 – D4 were amplified along with engineered EcoRI and XhoI sites. The forward primers for D1 (amino acids 1-56), D2 (amino acids 57-112), D3 (amino acids 113-168), D4 (amino acids 169-224) and D5 (amino acids 225-279) were *cggaattcaccatgagcctctttgatctcttcc, cggaattcaccatgccccctgaggaatttggc, cggaattcaccatggagacacctggtgagagac, cggaattcaccatgtttgatgatgtatggcctatg* and *cggaattcaccatggagcgccggactgtggtg* respectively. The reverse primers included *acgcgt cgacctagtgctgaggactatggaac* for D1, *ccgctcgagctatgactcaggacctggaag* for D2, *ccgctcgagctacct atgaaatggcctctgg* for D3, *ccgctcgagctactccactatcccatctgg* for D4 and *ccgctcgagctaccgggaccg gaaccaacg* for D5 respectively. The PCR products were gel-purified (1.5%), and cloned into the pGEX 5X-1 vector using EcoRI-Sal I sites for D1 and EcoRI-Xho I sites for D2-D5 of Hax-1 domains.

### pcDNA 3.1 (+) HA-Hax-1-D1-D5 constructs

For the construction of pcDNA3.1 (+) HA-Hax-1-D1 to D5 plasmids, 56 amino acid domains of Hax-1 were PCR amplified with primers containing RE sites for EcoRI and BamHI. Each of these constructs was engineered with a unique restriction site to distinguish from each other. The forward and reverse primers for D1 (amino acids 1-56) were *cggaattcagcctctttgatctcttcc* and *acgcggatcccccgggctagtgctgaggactatggaac* respectively, and contained SmaI *(cccggg)* as a unique restriction enzyme site. D2 (amino acids 57-112) contained Sal I *(gtcgac)* as a unique RE site, and had forward primer *cggaattcccccctgaggaatttggc* and reverse primer *ccgggatccgtcgacctatgactcaggacctggaag* for amplification. D3 (amino acids 113-168) construct was made using *cggaattcgagacacctggtgagagac* as a forward primer and *ccgggatccctgcagctacct atgaaatggcctctgg* as reverse primers with PstI (*ctgcag)* RE. D4 (amino acids 169-224 amino acids) was cloned out with *cggaattctttgatgatgtatggcctatg* forward primer and *ccgggatccgagctcctactc cactatcccatctgg* as reverse primer along with SacI *(gagctc)* as an identification site. Hax-1 D5 (amino acids 225-279) was PCR-amplified using *cggaattcgagcgccggactgtggtg* as forward primer and the *ccgggatccagtactctaccgggaccggaaccaacg* as reverse primer. The reverse primer for Hax-1 D5 included ScaI (*ctcgag*) as a unique identification site. The PCR products were gel purified (1.5% agarose gel) and cloned into the pcDNA 3.1(+) HA vector (Invitrogen).

### GST pull down Assay

The GST fusion proteins (GST-Hax-1-D1-D5) and the respective GST-fusion protein bound glutathione Sepharose 4B beads (GE Health Care) were prepared following previously published methods [48]. For the pull down assay, SKOV3 cells were lysed in a GST-lysis buffer containing 150 mM NaCl, 20 mM Tris (pH 8.0), 1mM MgCl_2_, 0.1% NP40, 10% glycerol incubated with the GST fusion protein domains D1-D5 of Hax-1 for 4 hours at 4°C, washed five times, and eluted in SDS sample loading buffer for analysis by western blotting.

### Confocal Microscopy

24 hours after transfection with the required constructs, 2 × 10^5^ transfectants were plated on to coverslips, fixed with 3% paraformaldehyde in PBS for 10 minutes, permeabilized with 0.1% Triton-X-buffer for 10 minutes and blocked for 30 minutes with ice cold 0.1% BSA in PBS. Sequential immunostaining was carried out with primary mouse monoclonal Rac1 (1:200) antibody for 1h, washed, incubated either with secondary Alexa 647-conjugated goat anti-mouse IgG (1:200) or Alexa 568-conjugated goat anti-mouse IgG for 1h, washed and then stained with Alexa 488-conjugated anti-mouse Cortactin (Millipore, MA) antibody for 1h. Nuclei were counter stained with DAPI (diamidino-2-phenylindole, 1μg/mL) for 5 minutes and washed finally with PBS. The coverslips were mounted on glass slides with 10 μl of Prolong Antifade reagent (Molecular Probes, Eugene, OR) and were analyzed with a Leica SP2 confocal microscope.

HA-epitope immunostaining in SKOV3 cells transfected with HA-tagged proteins was carried out using Alexa 647-conjugated mouse HA (Cell signaling, MA) antibody for 1h, counter stained with DAPI, mounted and analyzed as described above.

Transfection efficiency in the Hax-1 shRNA RFP construct transfected cells was determined after fixation with 3% paraformaldehyde, permeabilization with 0.1% Triton-X buffer and counter staining with DAPI. The coverslips were mounted as described above and micrographs were collected with a Nikon microscope. NIH ImageJ software was used to count the number of transfected cells.

All colocalization analyses were carried out using the JACoP (Just Another Colocalization Plugin) of NIH ImageJ software.

### Migration Assays

*In vitro wound healing assay:* The wound healing assay was carried out as described previously by our group [34]. 5 × 10^5^ cells were seeded into 60 mm culture dishes in 10% FBS media and allowed to adhere overnight. Cells were then washed three times with PBS and incubated in serum-deprived media for 24 hours. A linear scratch wound was made across the cell monolayer using the sharp end of a 200 μL sterile pipette tip (Sarstedt, Newton NC). The cells were washed with serum-free media to remove cellular debris. Fields of view (at 100 X magnification) were selected at random along the linear wounds and imaged using an Olympus CK40 microscope and Kodak DC290 camera system. The photographed fields were marked with a felt tip marker to allow re-identification at the next time-point. The cells were then incubated with serum-free media containing 20 μM LPA, or serum-free media alone for the control. After 24 hours incubation, the fields of view were identified and re-imaged.

*Transwell migration assay*: Cell migration was monitored using a transwell chamber assay as previously described [5, 34]. Cell culture inserts (polyethylene terephthalate membrane with 8.0 (m pores #353097, BD Biosciences, Franklin Lakes, NJ) were coated with rat-tail collagen, type 1 (BD Biosciences). 4 × 10^5^ cells in 200 (L serum-free media were placed in the well of the companion plate. The companion plate wells contained 500 (L of control serum-free media and serum-free media with 20 (M LPA or 10% FBS. The cells were incubated for 20 hours and the non-migrating cells on the proximal side of the inserts were removed with a cotton swab. The migrated cells on the distal side of the insert were fixed and stained with Hemacolor (EMD Chemicals, Inc., Gibbstown, NJ). The number of migrated cells was then enumerated with the images obtained from random fields of view at 10X magnification.

### Immunoprecipitation and Immunoblot analysis

Antibodies to cortactin (16-228) and Rac1 (05-389) were from Millipore (MA) whereas antibodies to hemagglutinin epitope (2362) and Hax-1 (H65220) were from Cell Signaling (Beverly, MA) and BD Biosciences (San Jose, CA), respectively. GAPDH antibody was from Ambion (4300). Peroxidase-conjugated anti-rabbit IgG (W401B and anti-mouse IgG (NA931V) were purchased from Promega (Madison, WI). 1mg of the lysate protein of the experimental groups were subjected to immunoprecipitation using the Rac1 or cortactin antibodies. The immunoprecipitates were resolved by 10% or 15% SDS-PAGE gels, and immunoblot analyses were carried out according to our previously published methods [5, 34, 49]. The blots were developed using Western Lightning Chemiluminescence Reagent (Perkin Elmer, Boston MA) and imaged using Kodak Image Station 4000 MM. Quantification of immunoblots was performed using Carestream Molecular Imaging Software version 5 (Rochester, NY) and the respective values were imported into Graph Pad Prism (La Jolla, CA) for graphing and statistical analysis.

## Abbreviations

Hax-1: HS1-associated protein X-1
HS1: Hematopoietic cell Specific protein 1
HCLS1: Hematopoetic Cell-specific Lyn Substrate-1 (HCLS1/HS-1)
FBS: Fetal Bovine Serum
LPA: Lysophosphatidic acid
RFP: Red fluorescent protein
